# Stabilization of myeloid-derived HIFs promotes vascular regeneration in retinal ischemia

**DOI:** 10.1007/s10456-019-09681-1

**Published:** 2019-10-03

**Authors:** Pilar Villacampa, Sidath E. Liyanage, Izabela P. Klaska, Enrico Cristante, Katja E. Menger, Robert D. Sampson, Maeve Barlow, Laura Abelleira-Hervas, Yanai Duran, Alexander J. Smith, Robin R. Ali, Ulrich F. O. Luhmann, James W. B. Bainbridge

**Affiliations:** 1 Division of Genetics, UCL Institute of Ophthalmology, University College London, 11-43 Bath Street, London, EC1V 9EL UK; 2 Present Address: Bellvitge Biomedical Research Institute (IDIBELL), Duran i Reynals Hospital, Gran Via de L´Hospitalet 199-203, 08908 Hospitalet de Llobregat, Spain; 3 Present Address: Roche Pharmaceutical Research and Early Development, Translational Medicine Ophthalmology, Roche Innovation Center Basel, F. Hoffmann-La Roche Ltd, Grenzacherstrasse 124, 4070 Basel, Switzerland

**Keywords:** Vhl, HIF, Myeloid cells, OIR, Vascular regeneration

## Abstract

The retinal vasculature is tightly organized in a structure that provides for the high metabolic demand of neurons while minimizing interference with incident light. The adverse impact of retinal vascular insufficiency is mitigated by adaptive vascular regeneration but exacerbated by pathological neovascularization. Aberrant growth of neovessels in the retina is responsible for impairment of sight in common blinding disorders including retinopathy of prematurity, proliferative diabetic retinopathy, and age-related macular degeneration. Myeloid cells are key players in this process, with diverse roles that can either promote or protect against ocular neovascularization. We have previously demonstrated that myeloid-derived VEGF, HIF1, and HIF2 are not essential for pathological retinal neovascularization. Here, however, we show by cell-specific depletion of Vhl in a mouse model of retinal ischemia (oxygen-induced retinopathy, OIR) that myeloid-derived HIFs promote VEGF and bFGF expression and enhance vascular regeneration in association with improved density and organization of the astrocytic network.

**Electronic supplementary material:**

The online version of this article (10.1007/s10456-019-09681-1) contains supplementary material, which is available to authorized users.

## Introduction

Myeloid cells play a key role in ocular neovascularization, which is a pathological feature of the common sight-threatening eye diseases retinopathy of prematurity (ROP), proliferative diabetic retinopathy (PDR), and age-related macular degeneration (AMD). Myeloid cells can either promote or protect against ocular neovascularization, depending on the timing of intervention and specific cell population. Depletion of macrophages by clodronate reduces retinal neovascularization in oxygen-induced retinopathy (OIR) and laser-induced choroidal neovascularization (CNV) [1, 2]. However, injection of myeloid precursors reduces retinal neovascularization and enhances vascular repair in OIR [3, 4]. We have previously shown that myeloid-derived VEGF production is not an essential requirement for ocular neovascularization [5], in contrast to other pathological conditions such as tumor growth [6]. Nor does deletion in myeloid cells of the genes encoding hypoxia-inducible factors HIF1α and HIF2α, which are strong activators of VEGF expression [7, 8], have an impact on ocular neovascularization [5]. Von Hippel–Lindau tumor suppressor protein (pVHL) targets HIF factors for rapid proteosomal degradation in normoxic conditions and in its absence their stabilization activates the HIF pathway [9, 10]. Both HIF1α and HIF2α isoforms are stabilized in the inner retina during the hypoxic phase of OIR with distinct cellular distributions [11]; despite their close structural homology, HIF1α and HIF2α act differentially during both development and hypoxia. Activation of the HIF pathway in astrocytes and neurons by deletion of *Vhl* is proangiogenic in the postnatal retina [12, 13], but in OIR systemic pharmacological activation of HIFs protects against retinal vasoregression and subsequent pathological neovascularization [14]. Here, we sought to determine the specific responses of myeloid cells to stabilization of HIF isoforms in retinal ischemia and to establish the impact on retinal vasculature. We did so by investigating OIR in mice with myeloid cell-specific deletion of *Vhl*, *Hif1a*, and/or *Epas1* (encoding HIF2α). We found that stabilization of both HIF1α and HIF2α in myeloid cells by *Vhl* deletion promotes expression of VEGF and bFGF and enhances retinal vascular regeneration in association with improved density and organization of the astrocytic network.

## Materials and methods

### Animals

Mice were used with institutional ethical approval and under a United Kingdom Home Office Project license and personal license. All procedures were performed in accordance with the Association for Research in Vision and Ophthalmology Statement for the Use of Animals in Ophthalmic and Vision Research. The following mice were used: *Lysm*^Cre/+^*Vhl*^flox/flox^ (*Vhl*^*MCΔ/Δ*^), *Lysm*^Cre/+^*Vhl*^flox/flox^*Hif1a*^flox/flox^ (*Vhl*^*MCΔ/Δ*^*Hif1a*^*MCΔ/Δ*^), *Lysm*^Cre/+^*Vhl*^flox/flox^*Epas1*^flox/flox^ (*Vhl*^*MCΔ/Δ*^*Epas1*^*MCΔ/Δ*^). All experimental genotypes used in this study were compared to age-matched littermate controls (non-Cre- expressing, flox/flox for the corresponding gene).

### Gene deletion assessment

Peritoneal macrophages were isolated 2 h after intraperitoneal injection of 30 ng lipopolysaccharide (LPS; Sigma, UK). CD11b+ cells were sorted (details below) in RTL buffer (Quiagen) and DNA/RNA was extracted using AllPrep DNA/RNA Mini Kit (Quiagen).

### OIR protocol

Mice were placed in hyperoxia at 85% O_2_ from P8 to P11, as previously described [15]. Nursing mothers were rested at room air for 2–4 h daily. Half of the pups of each litter were culled immediately following hyperoxia (P11) and the remaining pups culled 5 days later at the timepoint of maximum neovascularization (P16). Alternatively, pups were culled 2 days after the end of hyperoxia exposure (P13). Mice weights were recorded at culling; no significant differences were found (*Vhl*^*fl/fl*^ 7.9 ± 0.56 g, *Vhl*^*MCΔ/Δ*^ 8.1 ± 1.1 g). Mice weighing less than 5 g at P16 were excluded from the study.

### CNV protocol

CNV was induced with a diode laser as previously described [16]. Fundus fluorescein angiography by scanning laser ophthalmoscopy (Heidelberg Spectralis, Germany) was performed at 7 days and 14 days following laser CNV induction.

### Histology and image analysis

Eyes were enucleated and fixed in 4% paraformaldehyde for 1 h. After dissecting and blocking, retinas were incubated with biotinylated Isolectin B4 (Sigma-Aldrich) and Alexa Fluor 546-conjugated streptavidin (Life Technologies) and/or rabbit anti-GFAP primary antibody (Dako) and anti-rabbit-Alexa 488 (Molecular Probes) and then flat-mounted. Morphometric analysis was performed using Image J [17]. Avascular area was manually quantified and calculated as percentage of total retinal area; neovascular area was quantified as lectin-positive area in lesions, manually excluding normal vessel content, and calculated as percentage of total retina; healthy vascular area was calculated as total retinal area subtracting avascular area and neovascular area, and represented as percentage of total retinal area. In plots including different genetic models, avascular and neovascular area were normalized against their respective controls to control for strain-related variability. The astrocytic coverage (GFAP-positive area) was measured in whole retinas, excluding highly reactive edges and unspecific background using Threshold tool in Image J (ESM1). Values were calculated as percentage of the total retinal area measured (depicted in green). Sprout number and length, filopodia number, and myeloid positive cells were quantified in × 40 images and normalized by the extent of vascular front represented in the picture.

### FACS acquisition and cell sorting

Mouse retinas were dissociated into a single-cell suspension by using a papain neurosphere dissociation kit (Miltenyi Biotec, UK), according to the manufacturer’s instructions. Once dissociated, the samples were stained with a rat anti-mouse CD11b-BB515 antibody (BD Biosciences, USA) in DMEM^+^ media (2% FCS and 10 mM HEPES) for 30 min on ice, in the dark. The cells were then stained with SYTOX Blue Dead Cell Stain (2.0 μM final concentration) (Thermo Fisher Scientific, UK) and filtered through a 35-μM filter-capped tube (Falcon) just before cell acquisition. The samples were acquired and sorted on a 5-laser BD Influx cell sorter (BD Biosciences, USA) and collected in TRIzol plus (Thermo Fisher Scientific, UK) for RNA extraction.

### Quantitative PCR

RNA from cells sorted into TRIzol plus (Thermo Fisher Scientific, UK) was extracted using Direct-zol microprep RNA kit (Zymo Research, USA) and RNA from isolated retinas was extracted using the RNeasy mini kit (Qiagen). cDNA was made using the QuantiTect Reverse Transcriptase kit (Quiagen). qPCR was performed on an Applied Biosciences 7900HT thermocycler (Life Technologies) using the TaqMan probe-based PerfeCTa^®^ qPCR FastMix^®^ (VWR) with specific oligos for each gene.

### Statistical analysis

Data were analyzed using GraphPad Prism (Graphpad Software Inc.). Data in graphs are expressed as mean ± SEM. Unpaired Mann–Whitney’s t tests were used for comparing two unmatched groups and one-way ANOVA with Bonferroni correction for three or more unmatched groups. ***p < 0.001, **p < 0.01, *p < 0.05.

## Results

### Stabilization of myeloid cell HIF1s promotes retinal vascular regeneration

**Fig. 1:**
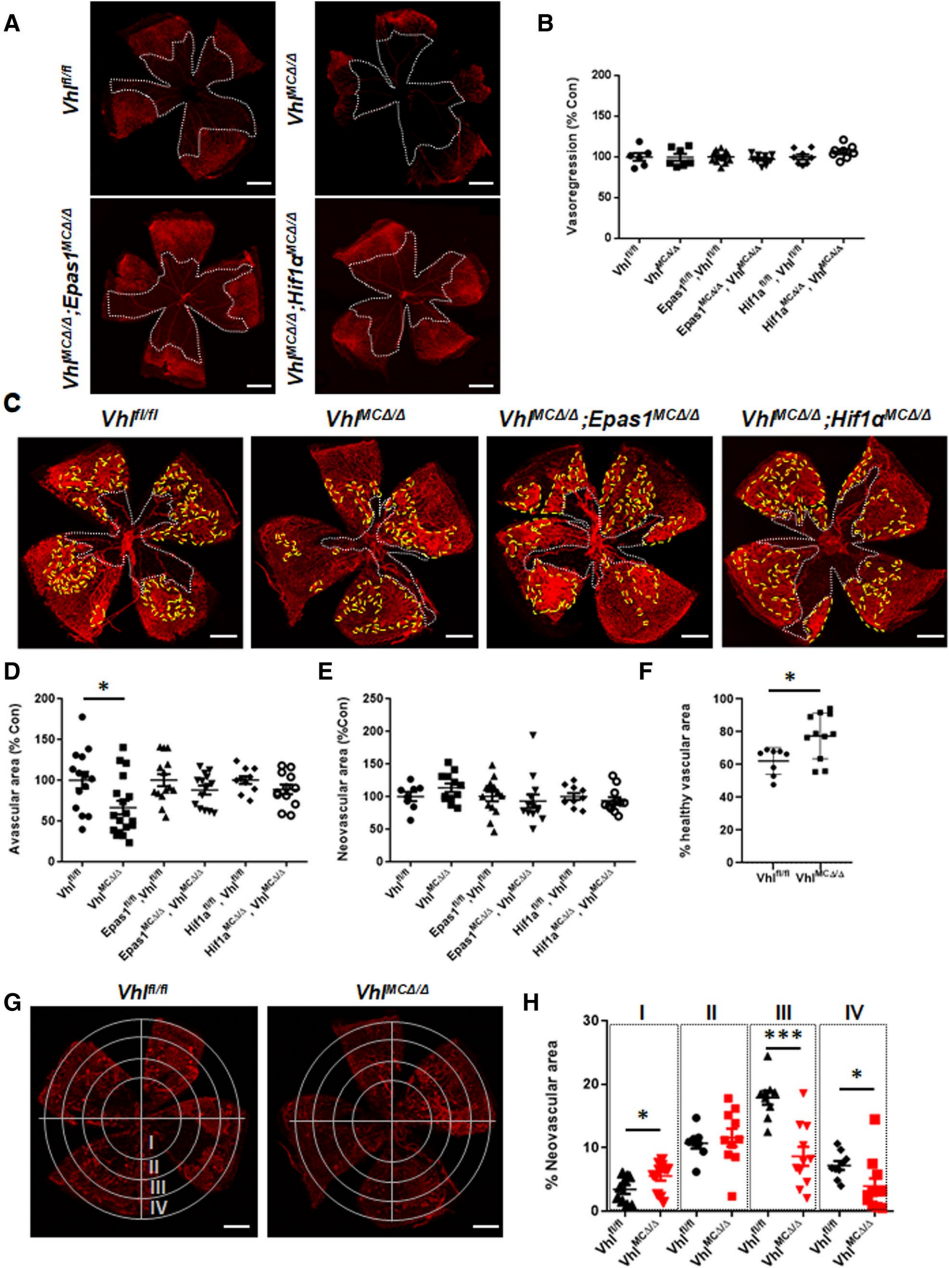
Stabilization of HIFs in myeloid cells promotes retinal revascularization in OIR. **a** Representative pictures of flat-mounted retinas from *Vhl*^*MCΔ/Δ*^*, Vhl*^*MCΔ/Δ*^*Epas1*^*MCΔ/Δ*^, and *Vhl*^*MCΔ/Δ*^*Hif1a*^*MCΔ/Δ*^ mice at P11 after hyperoxic exposure. **b** Retinal vasoregression (white depicted area) was similar in all the models after hyperoxic exposure (P11). **c** Representative pictures of flat-mounted retinas from *Vhl*^*MCΔ/Δ*^*, Vhl*^*MCΔ/Δ*^*Epas1*^*MCΔ/Δ*^, and *Vhl*^*MCΔ/Δ*^*Hif1a*^*MCΔ/Δ*^ mice at P16 after OIR. **d** Remaining avascular area was significantly reduced (by 35%) at P16 in retinas from *Vhl*^*MCΔ/Δ*^ mice but not in the double knock-out mice, although **e** the total neovascular area (yellow depicted area) remained unchanged in all the models. **f***Vhl*^*MCΔ/Δ*^ retinas showed increased (25%) healthy vascular area at P16 after OIR, when compared with control littermates. **g** Concentrical division of retinal area revealed **h** reduced neovascular area (~ 50% reduction) in the peripheral retinas from *Vhl*^*MCΔ/Δ*^ mice. Scale bars: 0.5 mm. *n* = 6–14 per group. Data are expressed as mean ± SEM. Values in **b**, **d**, and **e** are represented as % of control for each mouse model. Statistical analysis was performed by one-way ANOVA (**b**, **d**, **e**) and the two-sided Mann–Whitney test (**f**, **h**), *p < 0.05

We generated *Vhl*^*MCΔ/Δ*^*Epas1*^*MCΔ/Δ*^, *Vhl*^*MCΔ/Δ*^*Hif1a*^*MCΔ/Δ*^, and *Vhl*^*MCΔ/Δ*^ mice to stabilize only HIF1α, only HIF2α, or both HIF1α and HIF2α in myeloid cells, respectively. Downstream effectors of HIFs are upregulated in myeloid cells after *Vhl* deletion in these models [10]. All models showed effective deletion of floxed genes after Cre expression and normal retinal vasculature at postnatal day (P)16 (ESM2). We sought to determine the response of these models to oxygen-induced retinopathy (OIR). The extent of induced vasoregression after hyperoxia exposure was similar at P11 in all mutants and floxed littermate control animals (Fig. 1a, b). However, stabilization of both HIF1α and HIF2α in *Vhl*^*MCΔ/Δ*^ mice reduced the central avascular area at P16 by 35% (Fig. 1c, white depicted area, d), resulting in a 72% revascularization of the avascular area at P11 for mutant retinas, versus 57% for control retinas. Immunohistochemistry of retinal flatmounts showed that the number of sprouts, filopodia, and Edu + proliferating cells were similar in the vascular front of mutant and control retinas at P16 (ESM3a-b). No differences were observed in the extent of retinal neovascular areas at P16 (Fig. 1c, yellow depicted area, e) but greater healthy vascularized areas were evident in the periphery of *Vhl*^*MCΔ/Δ*^ retinas (25% of increment vs controls) (Fig. 1f), indicating improved vascular regeneration.

To investigate regional differences in retinal vasculature, we subdivided flat-mounted retinas for quantification into 4 concentric rings (Fig. 1g). We found no difference in overall pre-retinal neovascularization but identified differences in the topographical distribution of pre-retinal neovascularization in *Vhl*^*MCΔ/Δ*^ retinas at P16 (Fig. 1h). Myeloid-Vhl KO mice developed less neovascularization than control animals in the peripheral retina (rings III and IV, ~ 50% reduction) but greater neovascularization in the central area (ring I, 1.6-fold increase) (Fig. 1h). These results suggest that stabilization of HIFs in the myeloid cells results in a shift towards healthy revascularization in the early hypoxic phase.

To investigate the role of stabilization of myeloid-derived HIF1s in ocular neovascularization in adults, we induced choroidal neovascularization (CNV) by laser in adult mice of all genotypes. We observed no differences in the size of neovascular lesions at 7 or 14 days post-laser induction (ESM3c-d).

#### *Vhl*^*MCΔ/Δ*^ mice have delayed neovascularization and improved astrocytic template after OIR

**Fig. 2:**
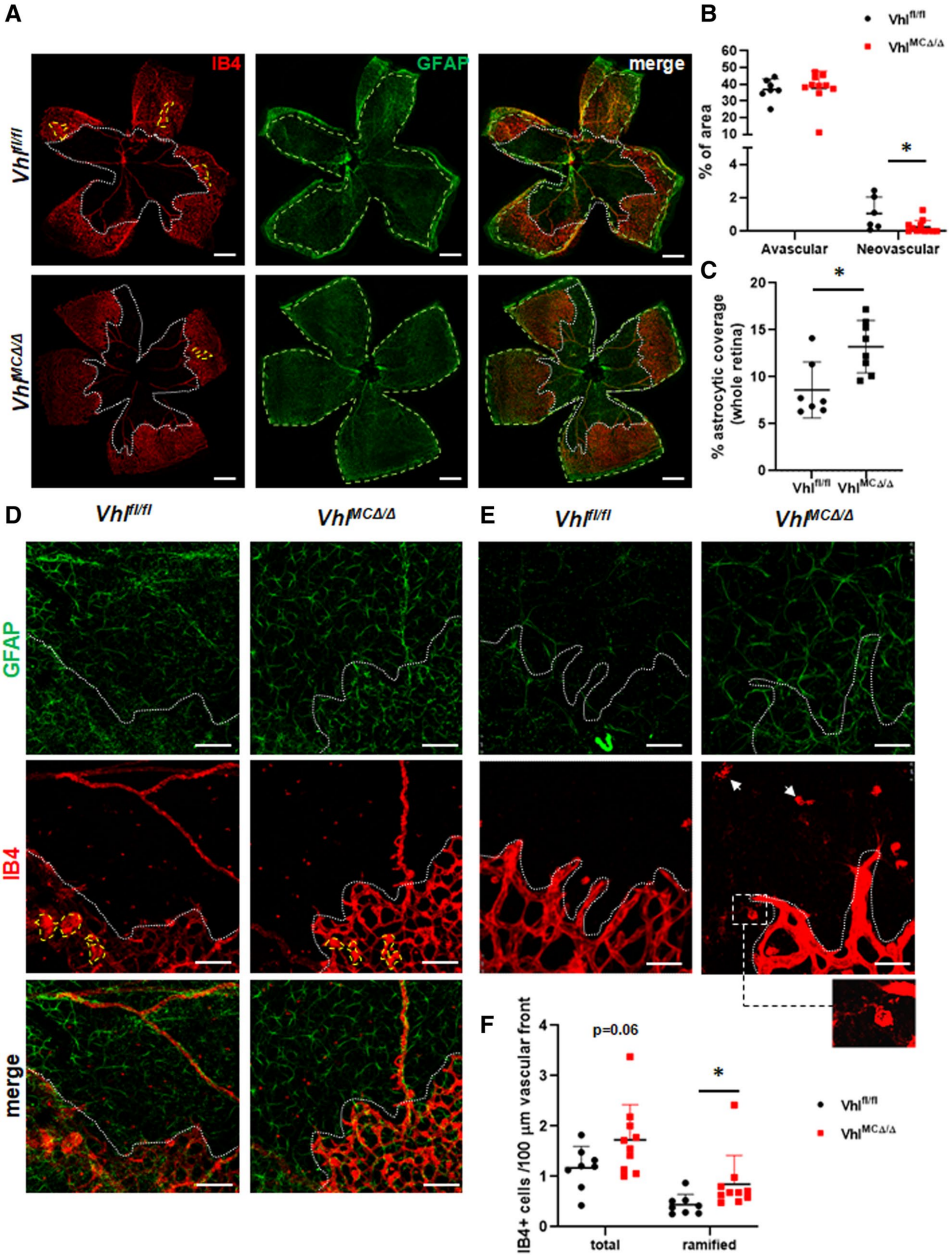
*Vhl* deletion in myeloid cells ameliorates neovascular responses in early OIR in association with improved astrocytic network. **a** Representative pictures of flat-mounted retinas from *Vhl*^*MCΔ/Δ*^ and control mice at P13 after OIR. **b** Reduced neovascular area (by 65%, yellow depicted area) was determined in *Vhl*^*MCΔ/Δ*^ retinas when compared with controls, whereas the avascular area remain unchanged (white depicted area). **c** Increased astrocytic coverage (GFAP-positive area) was detected in *Vhl*^*MCΔ/Δ*^ whole retinas at P13 after OIR (1.5-fold increase, quantified within green depicted area). **d**, **e** Higher magnification images showing improved density and organization of the astrocytic network in the avascular (**d**) and sprouting area (**e**) of *Vhl*^*MCΔ/Δ*^ retinas. Inset in **e** shows representative ramified, lectin-positive myeloid cell. **f** Non-activated, ramified myeloid cells were enriched in the sprouting front of *Vhl*^*MCΔ/Δ*^ retinas. Scale bars: 0.5 mm (**a**), 125 µm (**d**), 43.75 µm (**e**) *n* = 9–10 per group. Data are expressed as mean ± SEM. Statistical analysis was performed by two-sided Mann–Whitney test, *p < 0.05

To investigate further whether enhanced revascularization was occurring in the early neovascular phase in *Vhl*^*MCΔ/Δ*^ mice, we analyzed control and mutant retinas at P13, 2 days after their return to normoxia. At this timepoint, the extent of the avascular area (Fig. 2a, white depicted area, b) and the numbers of sprouts and filopodia (ESM3e) were similar in *Vhl*^*MCΔ/Δ*^ and floxed controls. However, the presence of neovascular tufts was reduced by 65% in *Vhl*^*MCΔ/Δ*^ retinas (Fig. 2a, yellow depicted area, b). Reduced neovascular responses in OIR have been associated with improved survival of both myeloid cells and astrocytes [18]. Accordingly, reduced neovascularization at P13 in *Vhl*^*MCΔ/Δ*^ retinas was associated with a 1.5-fold increased astrocytic coverage in the whole retinas of *Vhl*^*MCΔ/Δ*^ mutants (measured as GFAP-positive area, Fig. 2a,c; quantified within green depicted area), indicating a higher presence of astrocytes after hyperoxia exposure, a finding that may suggest improved survival of astrocytes after OIR. Better preservation of astrocytes and their organization was more evident in VO areas (Fig. 2d, e), as astrocyte depletion during OIR mainly occurs in those areas [19]. This improvement of the astrocytic template in the central avascular area of *Vhl*^*MCΔ/Δ*^ retinas was maintained at P16 (ESM4a-b). Interestingly, we measured a twofold increase in the number of ramified, non-activated myeloid cells at the vascular front of *Vhl*^*MCΔ/Δ*^ retinas at P13 (Fig. 2f, arrowheads and inset).

#### Retinal CD11b+ *Vhl*^*MCΔ/Δ*^ cells express increased VEGF and bFGF in OIR

**Fig. 3:**
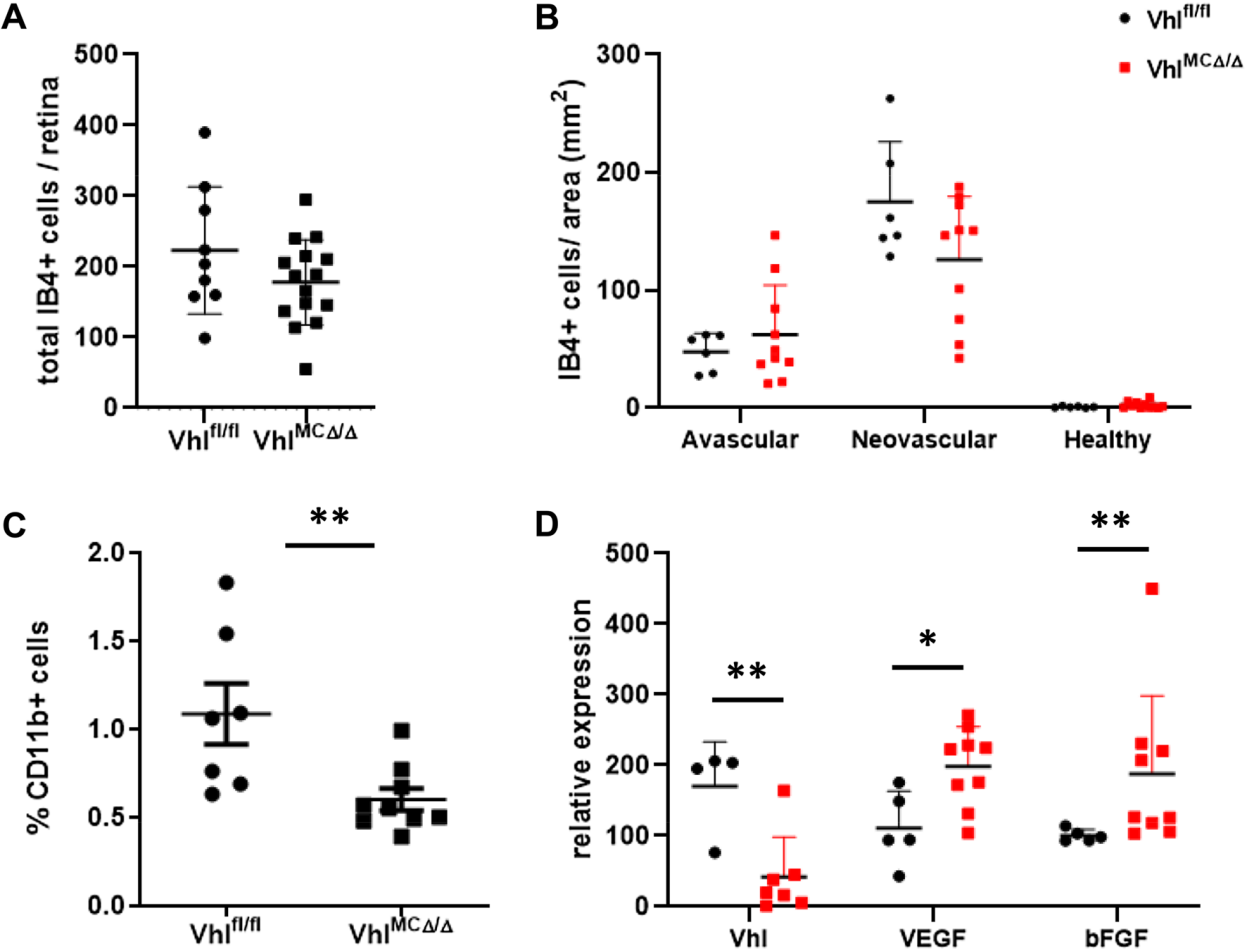
*Vhl*-deficient myeloid cells produced increased levels of VEGF and bFGF. Quantification of **a** total lectin-positive myeloid cells per retina and **b** lectin-positive myeloid cells per area revealed a trend for reduced numbers in neovascular areas of *Vhl*^*MCΔ/Δ*^ retinas. **c** Reduced population by 55% of CD11b-positive cells in *Vhl*^*MCΔ/Δ*^ retinas at P16 after OIR. **d** CD11b+ cells sorted from *Vhl*^*MCΔ/Δ*^ retinas showing depletion of Vhl, expressed more VEGF and bFGF (~ 1.8-fold increase) as measured by qPCR at P16 after OIR, when compared with cells sorted from control littermates. *n* = 4–15 per group. Data are expressed as mean ± SEM. Statistical analysis was performed by two-sided Mann–Whitney test, *p < 0.05, **p < 0.01

To determine the impact of Vhl deletion on retinal myeloid cells during ischemic retinopathy, we analyzed their distribution during OIR. At P11, no significant differences were observed neither in the total number nor in the distribution of myeloid lectin-positive cells (ESM4c-d). At P16, *Vhl*^*MCΔ/Δ*^ retinas showed a trend to have reduced numbers of myeloid positive cells (Fig. 3a). Cell distribution analysis revealed an enhancement of this trend specifically in neovascular areas (Fig. 3b, *p* = 0.09). This trend was confirmed by FACS analysis. *Vhl*^*MCΔ/Δ*^ retinas showed significantly fewer CD11b+ myeloid cells (55% reduction) at P16 after OIR compared with floxed littermates (Fig. 3c), consistent with lower levels of chemokine expression in *Vhl*^*MCΔ/Δ*^ retinas (ESM4e). CD11b+ sorted cells from *Vhl*^*MCΔ/Δ*^ retinas had significantly elevated expression of the HIF-dependent factors VEGF and bFGF (~ 1.8-fold change vs control cells) as determined by qPCR (Fig. 3d).

## Discussion

Retinal angiogenesis is a complex process that involves multiple cell types to build a vascular network sufficient to meet the uniquely high metabolic demand of retinal neurons. Uncontrolled vessel formation leads to pathological neovascularization, a common feature of many sight-threatening conditions. Myeloid cells have an active role in that process, acting as cellular chaperones to promote endothelial tip cell fusion during vessel formation and producing proangiogenic factors [20, 21]. HIF2α modulates HIF1α-driven proangiogenic responses by expressing sFlt1, stabilizing proliferating vessels, and promoting healthy revascularization [22]; HIF1 and HIF2 bind to different HREs in gene promoters, both being required for maximal transcriptional activity [23]. Here, we demonstrate that for myeloid cells only stabilization of both HIF1α and HIF2α improves vascular responses during ischemic retinopathy.

Retinas of *Vhl*^*MCΔ/Δ*^ mice were more effectively revascularized during the early hypoxic phase of OIR, although pathological neovascularization developed at a later timepoint. This effect of myeloid-specific Vhl depletion is not explained by increased vascular resistance to hyperoxia since the extent of oxygen-induced vasoregression was similar. We hypothesize that *Vhl*^*MCΔ/Δ*^ myeloid cells are “artificially” pre-adapted to hypoxia by established stabilization of HIFs, promoting early retinal revascularization more promptly and effectively in response to hypoxic conditions. This hypothesis is supported by the increased presence of non-activated, ramified myeloid cells in the vascular front of *Vhl*^*MCΔ/Δ*^ retinas at P13. This also may explain the absence of a similar response in the CNV model that is predominantly a model of tissue injury as opposed to hypoxia. Increased local production of VEGF and bFGF by mutant *Vhl*^*MCΔ/Δ*^ myeloid cells may also contribute to revascularization; injection of low doses of VEGF and bFGF reduces retinal neovascularization and promotes healthy revascularization in OIR [18].

Improved revascularization in OIR has been associated with improved survival of both myeloid cells and astrocytes [18]. Cross-talk between astrocytes and macrophages/microglia has been extensively studied in both physiological and pathological conditions [24]. *Vhl*^*MCΔ/Δ*^ retinas showed increased GFAP staining, suggesting either improved survival or function of astrocytes. Astrocyte-derived fibronectin matrix provides a template that guides sprouting ECs and regulates local VEGF availability to EC filopodia [25]. Although increased sprouting or proliferation was not detected at P13 and P16, our results suggest cooperation between astrocytes and myeloid cells in the formation of functional vasculature instead of pathological neovascular tufts. Our findings (Fig. 2) demonstrate a close spatial association of ramified myeloid cells, sprouting vessels, and an astrocytic network with improved coverage and organization.

Although *Vhl* deletion appears not to affect the trafficking of myeloid cells to the eye [10, 26], *Vhl*^*MCΔ/Δ*^ retinas showed significantly reduced number of CD11b+ cells. This may result from a milder phenotype of retinal neovascularization in the early phases of the hypoxic phase due to initial improved revascularization in *Vhl*^*MCΔ/Δ*^ retinas, with reduced drive to recruit CD11b+ cells to neovascular sites in OIR [27]. Although not statistically significant, our finding that *Vhl*^*MCΔ/Δ*^ retinas tended to have reduced lectin + myeloid cells density in neovascular areas (Fig. 3a) is consistent with this hypothesis.

We have previously demonstrated that myeloid-derived VEGF is not essential for pathological ocular neovascularization [5]. The findings of this study show that locally increased VEGF (and bFGF) expression by myeloid cells helps promote healthy vascular regeneration in the context of ischemic recovery. These findings support careful monitoring of VEGF-targeted therapies to avoid unwanted effects in vascular homeostasis due to excessive VEGF depletion.

## Electronic supplementary material

Below is the link to the electronic supplementary material.

**ESM 1** Astrocytic coverage quantification. **a** Whole retinal area was determined (dashed line) in GFAP immunostaining images, excluding highly reactive edges. **b** Example of GFAP green signal determination using Threshold tool in Image J in a flat-mounted retina. (PDF 139 kb)

**ESM 2** Effective gene deletion of **a** Vhl, **b** Hif1 or **c** Epas1 in peritoneal macrophages from *Vhl*^*MCΔ/Δ*^*, Vhl*^*MCΔ/Δ*^*Epas1*^*MCΔ/Δ*^ and *Vhl*^*MCΔ/Δ*^*Hif1a*^*MCΔ/Δ*^ mice after LPS challenge. **d** Normal retinal vascular development was observed at P16 in all the models. Scale bars: 0.5 mm. n=4-6 per group. Data are expressed as means ± SEM. Statistical analysis was performed by two-sided Mann Whitney test, *p<0.05. (PDF 509 kb)

**ESM 3 a,b** Number and length of sprouts and number of filopodia and Edu+ proliferating cells was similar in the vascular front of *Vhl*^*MCΔ/Δ*^ and control retinas at P16 after OIR. Choroidal neovascular lesions were similar at **c** day 7 and **d** 14 after laser induction. **e** Number and length of sprouts and number of filopodia was similar in the vascular front of *Vhl*^*MCΔ/Δ*^ and control retinas at P13 after OIR. *Scale bars: 25µm* (**a**)*. n=6-11 per group. Data are expressed as means ± SEM. Statistical analysis was performed by the two-sided Mann Whitney test (b,e) and one-way ANOVA (c,d).* (PDF 130 kb)

**ESM 4 a,b** More GFAP staining was present in the avascular area (dotted line) in *Vhl*^*MCΔ/Δ*^ retinas compared with control retinas. Quantification of **c** total lectin-positive myeloid cells per retina and **d** lectin-positive myeloid cells per area at P11 did not show any differences in *Vhl*^*MCΔ/Δ*^ retinas when compared with controls. **e** Expression levels of chemokines TNFα and MCP-1 in P16 retinas after OIR showed a tendency to be decreased in *Vhl*^*MCΔ/Δ*^ retinas. Scale bars: 100 µm. n=4-9 per group. Data are expressed as means ± SEM. Data are expressed as means ± SEM. Statistical analysis was performed by two-sided Mann Whitney test, *p<0.05. (PDF 935 kb)
