# Supplementary figures and images for: Stabilization of myeloid-derived HIFs promotes vascular regeneration in retinal ischemia
Source: Angiogenesis. 2019 Oct 3;23(2):83–90. doi: 10.1007/s10456-019-09681-1 (PMC7160070; doi:10.1007/s10456-019-09681-1)

ESM 1

A

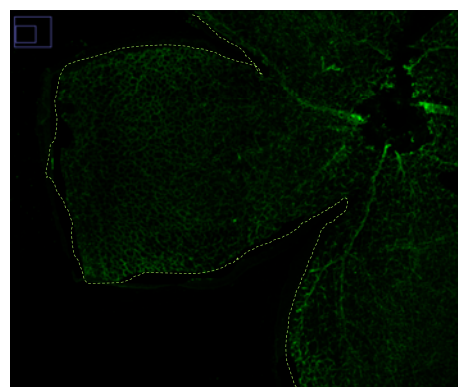

B

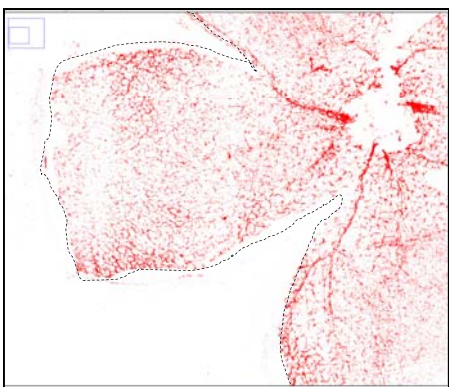

Supplement: Supplementary file 1 — ESM 1 Astrocytic coverage quantification. a Whole retinal area was determined (dashed line) in GFAP immunostaining images, excluding highly reactive edges. b Example of GFAP green signal determination using Threshold tool in Image J in a flat-mounted retina. (PDF 139 kb) [file 10456_2019_9681_MOESM1_ESM.pdf]

ESM 2

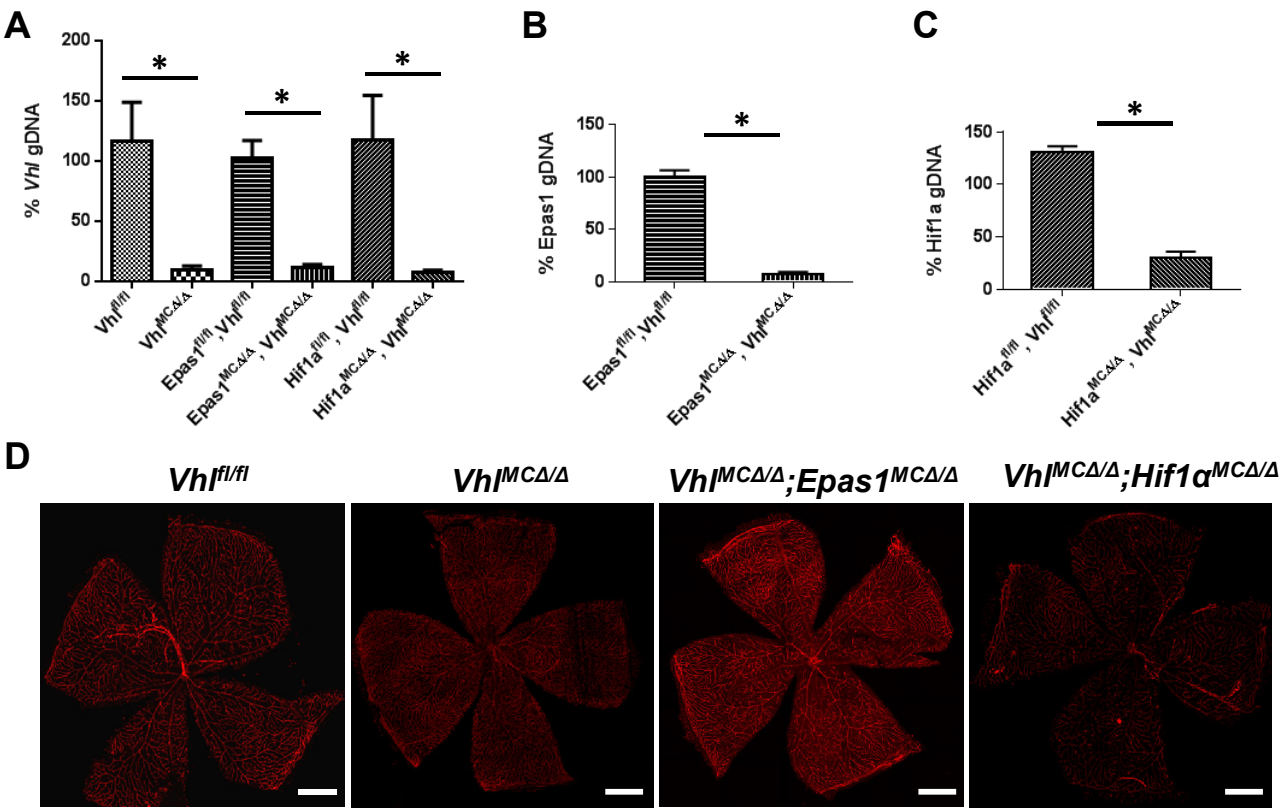

Supplement: Supplementary file 2 — ESM 2 Effective gene deletion of a Vhl, b Hif1 or c Epas1 in peritoneal macrophages from VhlMCΔ/Δ, VhlMCΔ/ΔEpas1MCΔ/Δ and VhlMCΔ/ΔHif1aMCΔ/Δ mice after LPS challenge. d Normal retinal vascular development was observed at P16 in all the models. Scale bars: 0.5 mm. n=4-6 per group. Data are expressed as means ± SEM. Statistical analysis was performed by two-sided Mann Whitney test, *p<0.05. (PDF 509 kb) [file 10456_2019_9681_MOESM2_ESM.pdf]

ESM 3

A

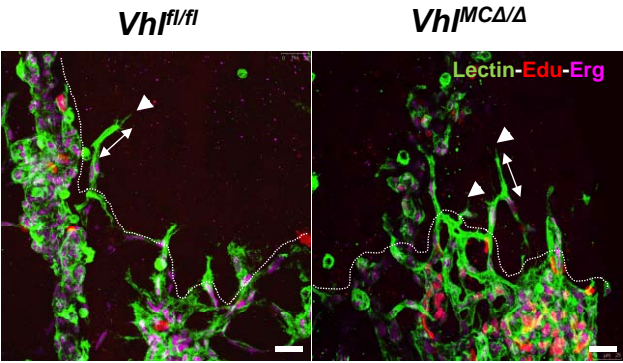

B

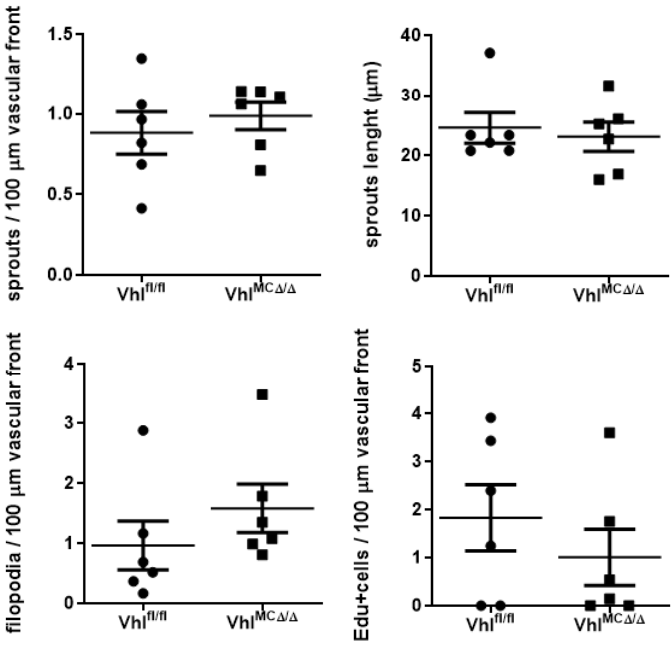

C

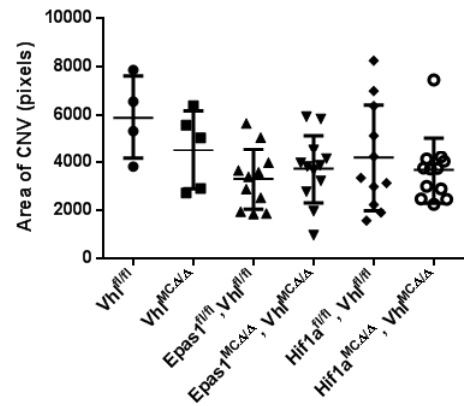

D

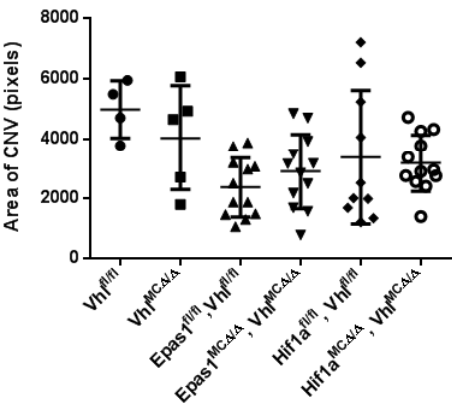

E

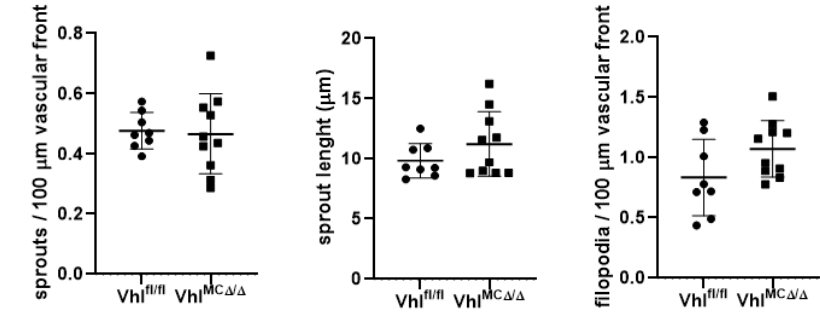

Supplement: Supplementary file 3 — ESM 3 a,b Number and length of sprouts and number of filopodia and Edu+ proliferating cells was similar in the vascular front of VhlMCΔ/Δ and control retinas at P16 after OIR. Choroidal neovascular lesions were similar at c day 7 and d 14 after laser induction. e Number and length of sprouts and number of filopodia was similar in the vascular front of VhlMCΔ/Δ and control retinas at P13 after OIR. Scale bars: 25µm (a). n=6-11 per group. Data are expressed as means ± SEM. Statistical analysis was performed by the two-sided Mann Whitney test (b,e) and one-way ANOVA (c,d). (PDF 130 kb) [file 10456_2019_9681_MOESM3_ESM.pdf]

ESM 4

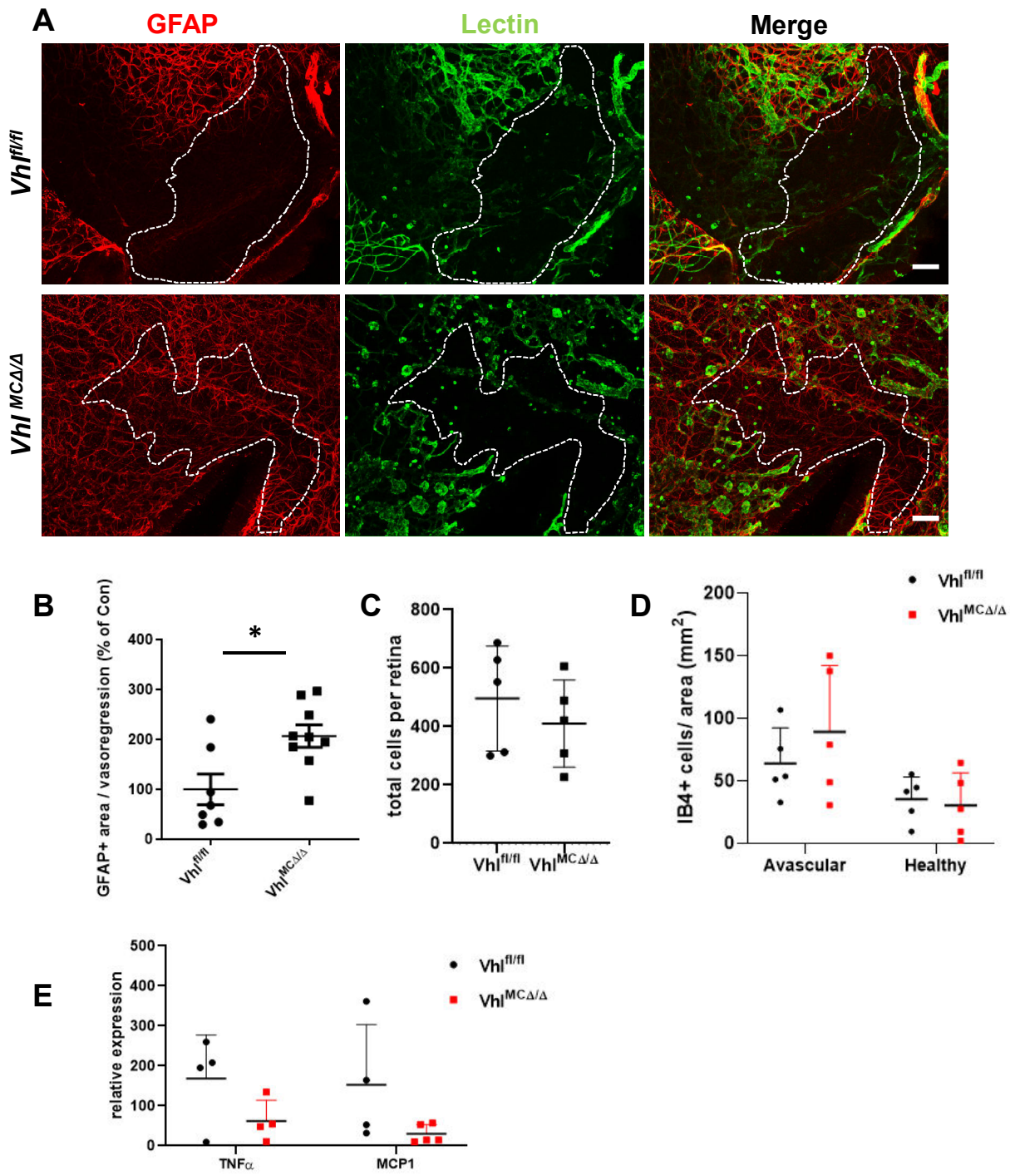

Supplement: Supplementary file 4 — ESM 4 a,b More GFAP staining was present in the avascular area (dotted line) in VhlMCΔ/Δ retinas compared with control retinas. Quantification of c total lectin-positive myeloid cells per retina and d lectin-positive myeloid cells per area at P11 did not show any differences in VhlMCΔ/Δ retinas when compared with controls. e Expression levels of chemokines TNFα and MCP-1 in P16 retinas after OIR showed a tendency to be decreased in VhlMCΔ/Δ retinas. Scale bars: 100 µm. n=4-9 per group. Data are expressed as means ± SEM. Data are expressed as means ± SEM. Statistical analysis was performed by two-sided Mann Whitney test, *p<0.05. (PDF 935 kb) [file 10456_2019_9681_MOESM4_ESM.pdf]
